# Concurrent evaluation of cerebral oxygen metabolism and upper airway architecture via temporally resolved MRI

**DOI:** 10.1177/0271678X251345293

**Published:** 2025-05-25

**Authors:** Jeffrey B Dennison, Michael C Langham, Andrew S Wiemken, Jing Xu, Richard J Schwab, John A Detre, Felix W Wehrli

**Affiliations:** 1 Department of Radiology, Perelman School of Medicine, 6572 University of Philadelphia, Pennsylvania, PA, USA; 2 Department of Sleep Medicine 6572 , Perelman School of Medicine, 6572 University of Pennsylvania, Philadelphia, PA, USA; 3 Information Management & Decision-making Science, Shanghai International Studies University, Shanghai, China; 4 Departments of Neurology and Radiology, Perelman School of Medicine, 6572 University of Pennsylvania, Philadelphia, PA, USA

**Keywords:** Brain oxygen metabolism, dynamic upper airway imaging, in-scanner EEG monitored sleep, obstructive sleep apnea, time-resolved MRI

## Abstract

Obstructive sleep apnea (OSA) disrupts the oxygen supply during apneic and hypopneic events. To evaluate the feasibility of concurrently monitoring cerebral metabolic rate of oxygen (CMRO_2_) and airway anatomy, a magnetic resonance imaging (MRI) pulse sequence was developed that interleaves measurements of CMRO_2_ with anatomic imaging of the upper airway at a temporal resolution of 5 seconds. The sequence was first tested in healthy subjects during wakefulness to detect the effect of volitional breath-hold and swallowing apneas on neuro-metabolic parameters and airway morphology. Subsequently, select patients with diagnosed OSA and healthy reference subjects were scanned during 90 minutes of wakefulness and sleep with concurrent electroencephalographic (EEG) monitoring and airway plethysmography. During non-rapid eye movement sleep, changes in metabolic parameters caused by neurovascular-metabolic uncoupling were detected, resulting in sleep-stage dependent reductions in the CMRO_2_. Spontaneous apneas were visible in airway images and confirmed plethysmographically. Recurrent apneas in patients during N1 and N2 sleep led to increased SvO_2_ and CBF (hypercapnic-hypoxic response) and decreases in SaO_2_ (hypoxemic response from airway closure) resulting in CMRO_2_ reductions as large 60%. The results demonstrate the MRI potential of noninvasive assessment of the dynamic changes in airway anatomy and brain metabolism in OSA during sleep.

## Introduction

Obstructive sleep apnea (OSA) is a sleep-related breathing disorder characterized by repeated narrowing or closure of the airway during sleep.^1^ The severity of OSA is expressed in terms of the hourly number of events of full or partial airway closures for at least 10 seconds (apneas and hypopneas, respectively) as the apnea/hypopnea index (AHI). The prevalence of the disease in the United States for moderate to severe OSA (AHI ≥ 15) is on the order of 10% among men but somewhat lower in women.^2^ Chronic sleep apnea adversely affects quality of life and predisposes patients to cardiac and neurovascular disease. OSA patients are also at elevated risk for neurological disorders, including Parkinson’s^3^ and Alzheimer’s diseases.^4^ Though the exact links between OSA and these comorbid neurological disorders are not fully understood, the oxidative stress resulting from recurrent hypoxia-reoxygenation during sleep has generally been regarded causative.^5^

To obtain detailed insights into the changes in brain metabolism in response to the recurrent cycles of hypoxia-reoxygenation associated with OSA, the key vascular-metabolic parameters — arterial and venous O_2_ saturation (SaO_2_ and SvO_2_) and cerebral blood flow (CBF) — must be measured during sleep when spontaneous apneas occur. However, there are a limited number of techniques for quantifying and localizing CMRO_2_ within the brain. Positron emission tomography (PET), based on the positron-emitting nuclide oxygen-15 (^15^O) as a tracer, has a long history as an accurate method for quantifying brain oxygen metabolism (see, for instance,^6^). However, the method is complex, costly, available at very few centers, and the long scan times preclude time-resolved quantification of O_2_ metabolism.^7^

During the past decade MRI-based methods relying on deoxyhemoglobin magnetism have emerged, allowing for non-invasive, rapid, calibration-free, quantification of CMRO_2_ and associated neurovascular and metabolic parameters (see recent reviews^8,9^). The target method uses the principles of susceptometry-based oximetry (SBO) to measure venous oxygen saturation in a major draining vein of the brain, typically the superior sagittal sinus (SSS), along with CBF.^10–12^ This method, referred to as OxFlow, or variants thereof, has been used in recent work to study the neurometabolic changes in OSA patients.^13–15^ Jensen et al. subjected OSA patients to hypoxia and found that they did not experience the significant reduction in CMRO_2_ experienced by healthy controls. Additionally, after CPAP treatment, the patients’ response normalized and became similar to healthy subjects. Two studies conducted in the present investigators’ lab with a dynamic variant of OxFlow found OSA patients had significantly lower baseline CMRO_2_ compared to healthy young subjects, but less so in age-matched controls.^15^ The same study simulated spontaneous apneas using repeated breath-hold stimuli after which participants experienced a large transient increase in CMRO_2_, confirming the hypercapnic-hypoxic nature of the stimulus. This transient increase in CMRO_2_ was exacerbated in the patients in comparison to non-OSA and young healthy subjects.

While cued apneas are a useful model to mimic spontaneous airflow disruptions, the mechanism underlying the latter is, of course, different from a volitional apnea induced via transient suspension of respiration. Further, since spontaneous apneas occur during sleep only, the neurometabolic changes during sleep must be taken into consideration. In recent work from the authors’ laboratory, the effect of sleep on oxygen metabolism has been investigated in healthy subjects sleeping in the scanner by running the high-speed OxFlow sequence with concurrent electroencephalographic (EEG) monitoring.^16,17^ The resulting data showed a reduction in CMRO_2_ which increased with sleep stage by up to 30% during N3 (slow-wave) sleep thought to be driven by neurovascular-metabolic uncoupling due to the reduction in synaptic transmissions^18^ during sleep. Thus, the response to spontaneous apneas in OSA patients is expected to be a superposition of two effects (sleep related decrease in O_2_ consumption and effect from transient hypoxemia) resulting from arterial desaturation. However, to correlate the cerebrovascular response with the transient upper airway anatomic changes during apneas it would be necessary to concurrently monitor airway anatomy and cerebral metabolism at appropriate frame rates to capture apneas typically ranging in duration from 10–30 seconds.

This paper introduces two technical advances of the basic OxFlow sequence to achieve the above objectives. First, a module for anatomic imaging of the upper airway (UA) was appended to the OxFlow sequence to observe and quantify the dynamic changes in upper airway morphology during apneas and hypopneas, permitting assessment of airway lumen and its association with changes in brain oxygen metabolism. Second, a spiral k-space sampling strategy was implemented in the velocity-encoded multi-echo gradient echo module designed for measuring blood flow velocity and induced phase in the superior sagittal sinus to obtain SvO_2_. The spiral encoding reduces acoustic noise from imaging gradient induced vibrations, which is crucial for enabling participants to achieve and maintain sleep. The superior spiral k-space sampling efficiency enhances temporal resolution, which is essential for measuring changes in oxygen metabolism associated with shorter apneic events, which can be as brief as 10 seconds.^19^ A reduction in slew rate and gradient amplitude during the anatomic imaging module further contributes to minimizing acoustic noise. The resulting dual-purpose pulse sequence, referred to as UA-OxFlow, partitions the scan cycle into an anatomic and vascular-metabolic portion, thus yielding structural information of the upper airway along with brain oxygen metabolic parameters at a temporal resolution able of capturing apneas.

We used the new method across two studies. The first is a validation experiment using cued apneas in a set of healthy participants to test whether we can observe the concurrent neurometabolic and upper-airway anatomic dynamics while yielding physiologically plausible values similar to those observed with recent variants of the parent OxFlow sequence.^11,12^ The second study was designed to detect the dynamic changes in neurometabolism and upper-airway morphometry during sleep in a set of healthy controls as well as OSA patients. Here, we projected to be able to replicate the previously observed neurovascular-metabolic decoupling in CBF and SvO_2_ leading to reduced CMRO_2_ in deeper levels of sleep^16,17^ as well as to detect apneas during sleep.

## Methods

### Summary of procedures

The protocol was approved by the University of Pennsylvania Institutional Review Board as guided by the ethical principles set forth in the Belmont Report. Written informed consent was obtained from all study participants before the examinations. To evaluate the method’s ability to detect apnea related changes in CBF, SvO_2_ and CMRO_2_, along with quantification of cross-sectional area of the upper airway, healthy participants performed a series of volitional apneas. The second study used simultaneous EEG and UA-OxFlow imaging on healthy participants and OSA patients during natural sleep. In this manner, disturbances in sleep related changes of neurometabolism as well as transient changes in neurometabolism in response to apneas and hypopneas could be detected and quantified. All participants were screened for standard MRI exclusion criteria (claustrophobia, pregnancy, metal implants, etc.) and were asked to consent to the procedure.

## Study protocol

### Model apnea study

The apnea paradigm was described to each participant as consisting of two sets of model apneas: a breath-hold apnea and a swallowing apnea defined as the initial phase of swallowing during which respiration ceases^20^ (i.e. the phase preceding the pharyngeal phase of deglutition). It is possible to maintain this phase for tens of seconds during which the upper airway is closed, thereby serving as a model for spontaneous apneas. Prior to entering the scanner, participants practiced the swallowing apnea maneuver and were instructed to “hold the apnea and attempt to breathe”. If they were unable to inhale during the model apnea, they were told they had successfully performed the swallowing apnea and instructed to follow the visual cues administered in the scanner via video screen prompts. Additionally, participants were instructed to lie still, and light cushioning was used to minimize involuntary head movement during the scan.

**Figure 1.**
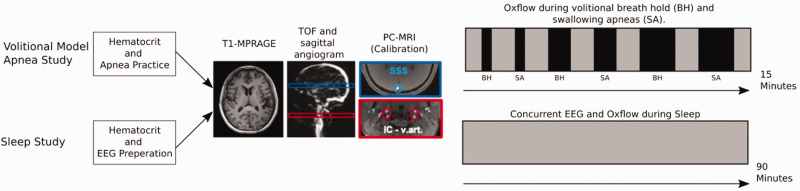
Timeline for model apnea and sleep study. Most elements are identical across both studies. Participants in the sleep study were fitted the EEG cap. The imaging procedure started with a time-of-flight angiogram to enable selection of slice locations for anatomic (airway) and the cerebrovascular-metabolic portion of the scan protocol detailed in Figure 2 below. MPRAGE images were collected to determine brain volume and mass, as CBF and CMRO_2_ are expressed in units scaled to 100 g of brain mass. In the model volitional apnea study, spiral UA-OxFlow was imaged for 15 minutes during which participants performed a series of increasingly longer breath-holds as described in the text. In the sleep study, UA-OxFlow was run for 90 minutes during which participants were encouraged to sleep.

The screen indicated an upcoming apnea event 22 seconds prior to its start, with the message remaining on the screen for 12 seconds. Participants were instructed to perform one cycle of inhalation and exhalation and to then initiate one of the model apneas. The apneic paradigm alternated between breath-hold and swallowing apneas and was comprised of three pairs each increasing in duration: 10 seconds, 20 seconds, and 30 seconds, separated from one another by 90 seconds each. The timeline of the paradigm is displayed in Figure 1. Hematocrit was measured using a portable hemoglobin meter (Hemocue Hb 201+, HemoCue America, CA, US). A digital pulse oximeter (Veris Medrad 8600, Bayer, PA, US), was attached to the participant's finger for continuous measurement of arterial oxygen saturation.

### Sleep study

Prior to arrival, all participants were asked to wake up early and to avoid caffeine to encourage in-scanner sleep. Each participant with OSA had previously been diagnosed and scored according to the AASM sleep manual. All participants were asked to arrive at the imaging center at 8:30–8:45 pm to allow time for informed consent and fitting and testing of EEG, SpO_2_, and nasal pressure canula monitors. Hematocrit was also taken at this time. After the participant was fit into the scanner, a series of calibration scans were completed, followed by a 90-minute period during which the participant was asked to attempt to sleep. Additional measures were taken to make participants comfortable and encourage sleep, including use of the larger 20-channel head-coil and appropriate cushioning, particularly beneath the knees. Light cushioning was also used to stabilize the head to minimize movement.

### MRI estimation of Whole-Brain CMRO_2_

CMRO_2_ was estimated based on Fick's Principle^10^ for both studies (model volitional apnea and sleep study), ignoring dissolved oxygen in plasma, which is less than 2%:

(1) $${\mathrm{CMRO}}_{2}=C_{a}\cdot \mathrm{CBF}\cdot \left({\mathrm{SaO}}_{2}-{\mathrm{SvO}}_{2}\right)$$

The hemoglobin carrying capacity of blood ( $C_{a}$ ) was estimated from the hemoglobin concentration obtained via a finger prick blood sample and the known value of 1.39 ml O_2_/g [Hb]^21^ for the oxygen content of fully saturated hemoglobin. Arterial oxygen saturation (SaO_2_) was measured via a pulse oximeter finger-probe. Both venous oxygen saturation (SvO_2_) and CBF are calculated from processing of the spiral Oxflow data as described below. Whole-brain CBF was quantified as detailed in Rodgers et al..^11^

The quantification of SvO_2_ within the SSS, which drains the cerebral cortex, is based on a measurement of the induced field resulting from paramagnetic deoxyhemoglobin in the vessel relative to the surrounding brain tissue by means of field mapping, as described previously^22^ given by:

(2) $${\mathrm{SvO}}_{2}=1-\frac{2\left|\Delta \phi \right|}{\gamma B_{0}\Delta \chi_{do}Hct\left(\cos^{2}\theta -1/3\right)\Delta TE}\times 100$$

Here Δφ is the average phase difference between intravascular blood and surrounding tissue accrued over a time ΔTE, γ is the proton gyromagnetic ratio, Δχ_do_ is the susceptibility difference between fully deoxygenated and fully oxygenated red blood cells (4π·0.27 ppm^23^) Hct represents hematocrit, and θ is the angle between the blood vessel and the static magnetic field B_0_, calculated post-hoc from the center of the SSS at two slice locations above and below the prescribed slice of the OxFlow sequence using 2 D time-of-flight angiograms.^24^

### EEG data acquisition, processing, and analysis

EEG acquisition and processing followed the analysis pipeline described in some of the present authors’ prior work.^16,17^ In brief, a 15-channel MR-compatible BrainCap, amplifier, and powerpack EEG system (Brain Products, Gilching, Germany) was used for collection of the EEG signal at a sampling rate of 5000 Hz. Electrode wells were filled with electrolyte gel after placement of the cap over the participant’s head and it was ensured that for each electrode the impedance was less than 10 kΩ. EEG data were analyzed using BrainVision Analyzer software (Version 2.1, Brain Products, Gilching. Germany), first correcting for MR gradient artifacts using the sliding average method^25^ (31 segments and followed by down-sampling to 500 Hz). Second, cardiobalistic artifacts were removed using an average ECG artifact template with independent component analysis (ICA) to further remove residual noise from the data and then submitted to band-pass filtering of 0.5–30 Hz before further down-sampling to 250 Hz.

EEG was then used to determine sleep stages, scored according to the American Academy of Sleep Medicine (AASM) criteria.^19^ Discrete 30-second intervals were each scored independently, and five sleep stages were identified and scored including, wakefulness (W), stage 1 (N1) of non-rapid eye movement (REM) sleep, NREM stage 2 (N2), NREM stage 3 (N3), and REM.

In one healthy reference subject a technical problem resulted in the disruption of the EEG system and a loss of 16 minutes’ worth of EEG data over the 90-minute scan. The 16 minutes of concurrent airway pressure and MRI data associated with this loss were removed from analyses.

### MRI data acquisition, processing, and analysis

MRI data were acquired at 3 T (Siemens Prisma, Siemens Medical Solutions, Erlangen, Germany) with subjects positioned head-first supine, using a 20-channel head-neck coil. A T_1_-weighted anatomical scan was taken to estimate brain volume and to normalize CBF and CMRO_2_ to 100 grams of neural tissue. Sagittal scout MR angiograms at the level of the SSS and neck were acquired to prescribe the locations for the CBF calibration scan and UA-OxFlow (see phase-contrast MRI flow velocity calibration, below). A midline sagittal image was prescribed through the center of the airway along with axial images at retropalatal and retroglossal airway locations (common sites of apneic airway closures) and oximetry at the straightest segment of the SSS.^24^

### Upper airway OxFlow pulse sequence

The spiral UA OxFlow sequence is a custom MRI sequence programmed using SequenceTree^26^ and consisting of two modules for upper airway anatomy and whole-brain oximetry.

**Figure 2.**
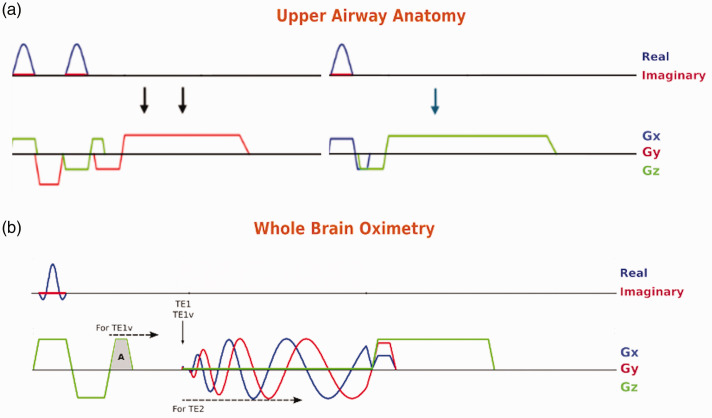
Upper airway spiral UA-OxFlow MRI pulse sequence. (a) Cartesian RF-spoiled GRE for anatomic imaging includes two neck slices acquired simultaneously with serial echo reformation technique, where two slices are excited back-to-back and resolved simply by dividing the k-space into two halves. The arrows indicate echo formations. Color codes indicate readout direction is along y-axis for necks and for the mid-sagittal, slice selective gradient is along x-axis with readout gradient along z-axis. For clarity, phase-encoding gradients are not shown but applied orthogonal to the slice-selection and readout gradients. Gradient spoiling was achieved by extending the readout gradient to help reduce acoustic noise and (b) for whole-brain oximetry, spiral OxFlow interleaves TE2, TE1 and TE1v (see text) at the level of SSS for computing field and velocity maps. The diagram shown corresponds to TE1. For TE1v, the third flow-compensating lobe, labeled A, is moved to the right for positive velocity encoding, m_1_ > 0. For TE2, the flow compensated spiral readout is delayed by 4 ms relative to TE1.

The anatomic images are interleaved and acquired with center-out ordered RF-spoiled gradient-recalled echoes (GRE) (Figure 2(a)). The OxFlow component interleaves between three center-out spiral RF-spoiled GRE (Figure 2(b)) consisting of two echoes with flow compensation (TE1) and velocity-encoding (TE1v) and one longer (TE2) with flow compensation. Field maps are generated with TE2 and TE1, while the velocity map is generated with TE1v and TE1. In short, UA spiral OxFlow is designed by concatenating Cartesian and spiral GREs. The primary rationale for using spiral readout was to reduce acoustic noise and acquisition time. Further reduction in acoustic noise was achieved by using the time saved from spiral readout to lower slew rate and gradient amplitude in anatomic imaging. The following imaging parameters were used for UA: TE/TR = 2.8/13.08 ms, flip angle = 11°, FOV necks = 192_x_ × 192_y_ mm^2^, matrix necks = 160 × 120 (partial Fourier factor = 0.75), FOV sagittal =384_z_ × 192_y_, matrix sagittal = 320 × 120 (partial Fourier factor = 0.75), max slew rate = 0.1 [mT/m]/µs, max gradient amplitude = 10 mT/m, bandwidth/pix = 312.5 Hz. The imaging parameters of constant linear speed spiral OxFlow were as follows: TE1/TE1v/TE2/TR = 4.75/4.75//8.75/20.01 ms, flip angle =10°, FOV = 224 mm, matrix = 448 (grid size factor = 2), number of shots = 31, k_max_ = 500 m^−1^, bandwidth/pix = 164 Hz, max gradient amplitude = 26.25 mT/m, max slew rate = 0.1178 [mT/m]/µs. The acquisition times for anatomic images and spiral OxFlow are 3.14 s and 1.86 s, respectively, i.e. the total acquisition time is 5 s.

### Phase-Contrast MRI flow velocity calibration

Flow rates at the SSS were obtained by multiplying blood flow velocity by the vessel’s cross-sectional area. Since the SSS accounts for only 45–55% of total brain blood flow, both whole-brain and SSS CBF were collected for a brief period while the subject was awake. In this manner the ratio of total CBF to SSS-CBF (referred to as “upscale ratio”) could be determined. During the 90 minute time series only SSS-CBF data are collected (to achieve the desired temporal resolution of 5 seconds). SSS-CBF then was subsequently upscaled to obtain whole-brain CBF for each time point. This approach, originally described in ref. 11, has been validated by Caporale et al, who showed that the upscale ratio obtained during wakefulness remains constant during sleep.^27^

### Processing of MRI data

The raw data was saved and processed offline using a set of in-house MATLAB scripts (MATLAB R2016b MathWorks, Inc., Natick, MA). The partial-Fourier anatomic data were sinc-interpolated before performing 2 D Fourier transformation. The multi-channel data was combined via sum-of-squares and a 2 × 2 median filter was applied to the magnitude images to enhance edge definition and reduce noise. For re-gridding the fully sampled spiral data, the density compensation function was computed using the vector approach,^28^ where the gradient waveform was calculated from the target k-space trajectory. Finally, density compensated data was convolved with a Kaiser-Bessel gridding kernel (window width of 3 and over grid factor of 2), as described in.^29^ Discrete Fourier transform was then performed on the re-gridded data. The resulting complex images were used to generate velocity and field maps by taking the phase difference between TE1v and TE1 and between TE2 and TE1, respectively. Lastly, data was visually assessed frame by frame, and sections of the time-series data showing motion corruption, were excluded from analysis.

## Statistical analysis

### Model apnea study

A generalized linear model was fit to test the effect of each volitional model apneas on CBF, SvO_2_, CMRO_2_, and cross-sectional airway. The presence of each apnea was modeled separately as a 1 or 0 and convolved with a single gamma to represent the hemodynamic response function.^30^ Additionally, before fitting the model, data was pre-whitened to reduce the impact of temporal autocorrelation on the analyses^31^ and better estimate error. This allows us to study the whole period of model apneas without suggesting immediate responses in neurometabolic parameters and to compare shorter and longer breath holds.

### Sleep study

As this study is primarily designed to determine the feasibility of a novel MRI method during sleep, we predominantly report descriptive statistics of feasibility outcomes in both healthy reference subjects and OSA patients. Specifically, we quantified the number of apneas and hypopneas observed by MRI and airway plethysmography in relation to transient changes in CMRO_2_. In order to evaluate sleep-dependent neurometabolic changes in OSA patients, a mixed-effects ANOVA model was run. First, the main effect of sleep stage was tested for CMRO_2_ from lighter to deeper stages of sleep (awake > N1 > N2 > N3), followed by testing the interaction of clinical status (OSA versus healthy reference) on the relationship between sleep-stage and CMRO_2_. In order to avoid data corruption by subject movement correlated noise, such epochs were excluded from the analysis.

## Participants

### Model apnea study

Seven participants (mean age: 28 years, SD: 6.21 years; 57.1% male) were recruited for the model apnea study (see Table 1(a) for details).

**Table 1.** Demographics for the participants in the model apnea (Study 1) and sleep study (Study 2).

(a)			
Study 1: Volitional Model Apneas			
Participant ID	Age (years)	Sex	BMI
101	52	M	24.3
102	24	F	25.7
103	26	M	40.7
104	26	F	21.3
105	24	M	21.5
106	33	M	26.5
107	22	F	20.3
(b)			
Study 2: Sleep Study			
Participant ID	Age (years)	AHI	Group
HR-201	26	N/A	Healthy Reference
HR-202	24	N/A	Healthy Reference
HR-203	24	N/A	Healthy Reference
HR-204	26	N/A	Healthy Reference
OSA-205	65	45.7	OSA
OSA-206	47	32.3	OSA
OSA-207	59	40.4	OSA
OSA-208	52	35.7	OSA
OSA-209	53	50	OSA
OSA-210	36	29.3	OSA
OSA-211	56	46	OSA

### Sleep study

A total of seven OSA patients (mean age: 51.86 years, SD: 8.46 years; 85.7% male) and four healthy reference participants (mean age: 25 years, SD: 1 years; 75% male) were recruited for the sleep study (see Table 1(b) below for details). Patients who were on CPAP agreed to undergo a “washout” period consisting of interrupting wearing the mask for 48 hours prior to the study, so as to reveal their OSA symptoms in the absence of treatment.

## Results

### Model apnea study

**Figure 3.**
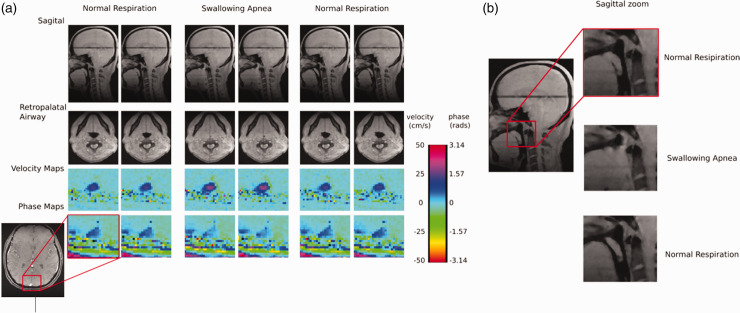
Sample images from the spiral UA-OxFlow sequence run during normal respiration and swallowing apnea challenge in a healthy test subject. (a) Anatomic images of the upper airway (sagittal, top, transverse, bottom). Note that across these images the airway is no longer visible during swallowing apnea. Bottom rows (color) are enlarged parametric images of superior sagittal sinus velocity and phase as indicated. Note enhanced blood flow and reduced phase (due to increased SvO_2_) during the apneic challenge and (b) magnified sagittal views of the upper airway allowing visual confirmation of airway closure during swallowing apnea versus normal respiration.

Figure 3 displays the anatomic images of the upper airway, one sagittal and one transverse, as well as one velocity and one field map, which were reconstructed for each 5-second epoch. During (volitional) model apneas we can clearly see the complete closure of the airway in the anatomic images and increased blood flow velocity in the SSS, along with a decrease in phase resulting from the transient reduction in oxygen extraction (i.e., increase in SvO_2_), consistent with the hypercapnic response to the apneic stimulus.

From these images estimates were obtained for CBF, SvO_2_, CMRO_2_, and cross-sectional area of the upper-airway for each successive 5-second epoch, thus creating a time-series. Average baseline values for CBF (53 ± 5 [mL/min]/100g), SvO_2_ (72 ± 4%), and CMRO_2_ (125 ± 23 [μmol O_2_/min]/100g), were comparable to those found in earlier studies by OxFlow, 48.6 ± 7 (mL/min)/100 g for CBF, 68.6 ± 3.0% for SvO_2_, and 125.1 ± 11.4 [μmol O_2_/min]/100g for CMRO_2_.^11^

**Figure 4.**
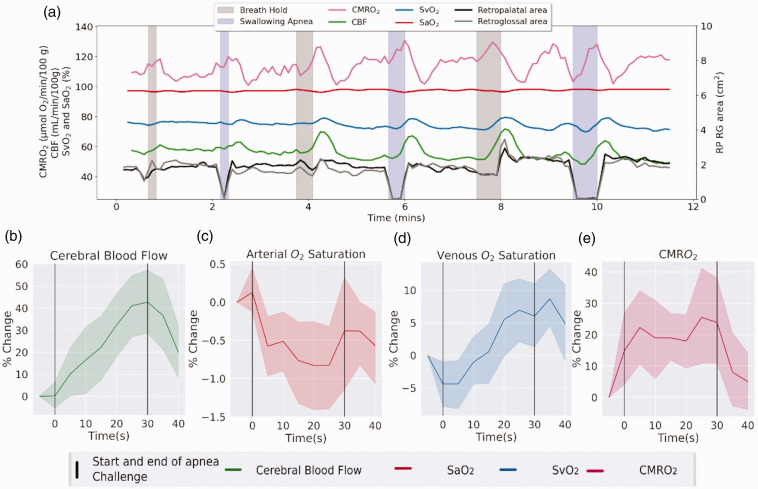
Dynamic changes in neurometabolic parameters and upper-airway anatomy in response to experimental apneas. (a) Changes in cerebral oxygen metabolism and upper airway architecture over the experimental time-course in one subject. Average changes across subjects in the neurovascular metabolic parameters, as indicated in panels (b)–(e), i.e., enhanced CBF and SvO_2_, along with a reduction in SaO_2_, resulting in a small but significant increase in CMRO_2_ in response to transient airway closure.

The presence of each apnea was modeled via a single gamma hemodynamic response function.^30^ and pre-whitened to reduce temporal autocorrelation.^31^ During apneas CBF increased from a baseline of 52.7 to a peak of 73.63 [mL/min]/100g (+40%; p < 0.001) while SvO_2_ increased from a baseline of 71.6 to 77.4% (+8.1%; p = 0.03). As CMRO_2_ is derived from SvO_2_ and CBF, these changes resulted in an increase of average CMRO_2_ from a baseline of 124.6 to a peak of 150.2 [μmol O_2_/min]/100g (+20%; p < 0.001) along with a decrease in SaO_2_ from 98.1 to 97.1% (−1%; p < 0.001) (Figure 4). An additional test of the cross-sectional area of the retro-palatal location was used to determine if there was a preferential response to swallowing as compared to the breath-hold apnea. To test this hypothesis another generalized linear model was run with two regressors representing the timing of either the swallowing or breath-hold apnea predicting the cross-sectional area of the upper airway. A significant interaction between apnea types (F (7,1) = 11.17; p = 0.012) indicated that the architecture of the upper airway was preferentially susceptible to the swallowing apnea. Separately modeling the apneas, we found that while the swallowing apnea paralleled consistent decreases in the cross-sectional area of the upper airway from an average of 0.8 cm^2^ to 0.05 cm^2^ (−93%; p < 0.001), the breath-hold apnea did not (p = 0.57). Together these results suggest that UA-OxFlow can identify specific airway narrowing or closures and neurometabolic changes during volitional model apneas.

### Sleep study

**Figure 5.**
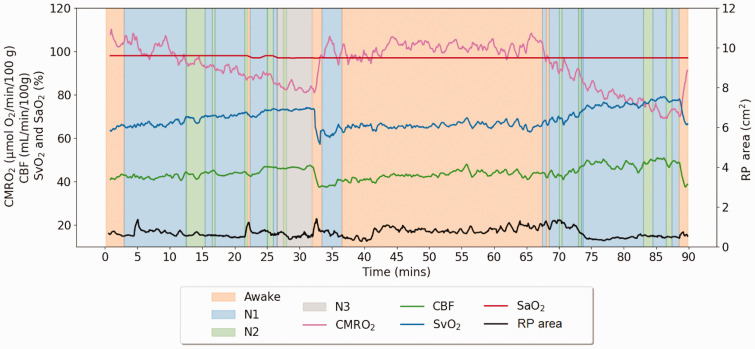
Representative time-series of neurometabolic information during wake and sleep from a healthy 24-year-old woman (ID HR-202). The data exhibit significant fluctuations in the cross-sectional area of the retropalatal (RP) upper airway during sleep, but no apneas. CMRO_2_ drops steadily with increasing periods of sleep, primarily as the result of increase in SvO_2_ due to reduction in O_2_ extraction, to a nadir of about 80 (μmol O_2_/min)/100 g during stage 3 sleep. Conversely, as the subject arouses, CMRO_2_ increases and this general pattern during a second sleep cycle toward the end of the scan repeats itself.

The feasibility of conducting studies of oxygen metabolism during a sleep-study was evaluated with respect to the method’s ability of promoting participant sleep in the scanner, linking temporal changes in neurometabolism to differing levels of sleep and apneic events, and lastly, comparing the sleep-stage related decrements in CMRO_2_ with prior data in the laboratory obtained with the parent single-level OxFlow sequence.^17^ Of the 10 participants enrolled all were able to tolerate the procedure for the full duration of the study and all were found to have achieved N1 sleep, with nine reaching at least N2 sleep. Of the four healthy reference subjects, three achieved N3 slow-wave sleep, as did two of the OSA participants. These results suggest that the current protocol enables participants to achieve multiple levels of sleep. In comparison to an earlier protocol based entirely on Cartesian encoding, the spiral encoding used in the module designed to quantify SvO_2_ and CBF (see Figure 2), acoustic noise was attenuated by 10 dB in the audible spectrum, thereby substantially improving the probability to achieve sleep. Figure 5 shows the time-course of vascular-metabolic parameters and retropalatal cross-sectional area in a healthy subject.

Additionally, feasibility requires that apneas occur during the scan period so their effects can be observed. Of six OSA patients, five experienced one or multiple apnea events. This suggests that the 90-minute sleep session is of a duration comparable to a full sleep cycle and is sufficient for capturing the transient changes in neurometabolism during apneas. During an apnea event, airflow measured via a nasal canula decreases and can be corroborated from the sagittal anatomic images returned by UA-OxFlow. The relationship between the occurrence of these apneas can then be related to the changes in cross-sectional area at the measurement location in the upper airway, CBF, SvO_2_, and CMRO_2_.

**Figure 6.**
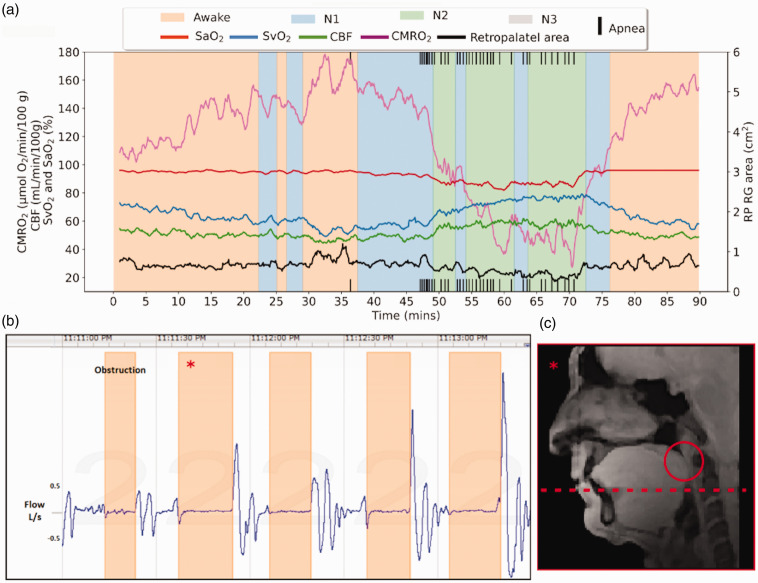
Data from a representative OSA patient (ID OSA-201). (a) Neurometabolic time-series obtained from a 54-year-old male with diagnosed sleep apnea, displayed with overlaid carpet plot indicating the occurrence of apneas. The patient’s CMRO_2_ decreases slowly following onset of sleep. At ca. t = 47 min, CMRO_2_ sharply falls, reaching a nadir of about 40 (μmol O_2_/min)/100 g, resulting from rapid increase in both CBF and SvO_2_ consistent with hypercapnia. (b) Plethysmographic recording of a series of closely spaced apneas showing complete loss of air flow during each of five successive events occurring over a period of about two minutes and (c) sagittal airway image corresponding to the second apnea (asterisk) and contact between the retropalatal boundary and posterior airway margins. The axial position (red dashed line) for the location chosen prior to scanning (indicated by the dotted red line) is below the site of airway closure, explaining why the retropalatal cross-sectional area plotted in Figure 6(a) does not reach zero during retropalatal airway closure.

Figure 6 shows time-course data for neurometabolic and airway structural parameters in a patient with established OSA. The patient, a 64-year-old male, has been on CPAP treatment for several years but was willing to undergo a 48-hour “washout” period to allow temporary return of symptoms prior to undergoing the protocol. He was scanned with a precursor of the current UA-OxFlow sequence displayed in Figure 2, differing only in the anatomic portion of the scan sequence that used Cartesian instead of the current spiral encoding. The data in Figure 6(a) show a large increase in SvO_2_ and a somewhat lesser increase in CBF during N2 sleep, along with significant arterial desaturation. The combination of these transient parameter changes explains the very large drop of over 50%, in CMRO_2_ (see Fick’s Principle equation). It is this period (transition to and during N2 sleep) during which most apneas occur. A string of five successive apneas occurring over a period of approximately two minutes are corroborated by the concurrently recorded airway plethysmograms in Figure 6(b), indicating total airway closure. Figure 6(c) shows one of the concurrently recorded sagittal images indicating retropalatal airway collapse. Of note is that the slice locations chosen for the axial anatomic images (which must be selected prospectively, without prior knowledge of the site of airway collapse) were slightly below the site of obstruction, and therefore the airway cross-sectional area plotted in Figure 6(a) does not reach zero during apneas. The overall pattern exhibited by this patient (OSA-205, Table 1(b)) is seen in other patients with moderate to severe apnea (see Fig. S1 in Supplemental Materials section).

To compare sleep-stage dependent changes in CMRO_2_ with some of the authors prior work,^16^ a mixed methods model with a within-subject effect of sleep stage, and a between-group effect of clinical status, was examined. The data revealed a significant main effect of sleep stage, suggesting a decrease in CMRO_2_ from average awake values by 2.7 ± 0.3%, (p < 0.001) which was associated with each stage of deeper sleep achieved in healthy subjects (Fig. S3). A significant interaction between sleep stage and clinical status suggested that OSA patients exhibited an additional decrease of 4.3 ± 0.5% from awake baseline for each stage of deeper sleep beyond the changes experienced by healthy controls (p < 0.001). These results suggest that patients with OSA tend to experience a steeper decline in CMRO_2_ as they enter deeper levels of sleep than does the healthy reference group. Identifying at which stage those differences become significant will require a larger sample and more participants who enter deeper levels of sleep.

## Discussion

A number of studies have been reported recently in which MRI was used to differentiate OSA patients from reference subjects, both in terms of baseline measurements or in response to stimuli in the form of cued breath-holds mimicking apneic events,^15,32^ or externally administered hypercapnia^33^ or hypoxia.^14^ Two of these studies show that cerebrovascular reactivity was elevated in apneics.^15,33^ The work by Rodgers et al.^11^ and Wu et al.^32^ showed depressed CMRO_2_ at baseline and enhanced transient elevation of CMRO_2_ following breath-hold stimulation. However, while some of these stimuli resemble the physiologic response to apnea, they differ from spontaneous apneas in several respects, most importantly, in that spontaneous apneas occur during sleep only.

The present work introduced a new quantitative MRI method that can assess brain oxygen metabolism at a temporal resolution of 5 seconds during sleep in the scanner, simultaneously with collection of anatomic information of the upper airway and concurrent EEG monitoring, targeting patients with obstructive sleep apnea. Toward this goal, we designed and implemented an MRI imaging pulse sequence, denoted UA-OxFlow, standing for upper-airway OxFlow, that partitions the image data acquisition cycle into two parts in which anatomic information is collected from two axial planes at retropalatal and retroglossal location, along with a midline sagittal image, followed by a second module collecting blood-flow and oxygen metabolic information yielding whole-brain venous oxygen saturation, together providing CMRO_2_ in a time-series mode during sleep in the scanner.

In healthy participants performing model volitional apneas in the form of breath-holds (BH) and swallowing apneas (SA), the latter representing an apneic stimulus that more closely resembles airway closure during spontaneous apneas, consisting of volitional extension of the initial phase of swallowing. Both types of induced apneas elicited a similar vascular-metabolic response, i.e., a transient increase in CBF, SvO_2_, and CMRO_2_, confirming the hypercapnic-hypoxic nature of temporarily disrupted airflow to the lung. Importantly, the sequence enables visualization and quantification of temporal changes in upper airway architecture, airway closure as short as ten seconds.

Healthy participants and a sample of OSA patients were scanned in a separate, second study, while running UA OxFlow for 90 minutes during which subjects transitioned from wakefulness to various sleep stages. MRI scanning was synchronized with airway plethysmography enabling establishment of ground truth for apnea detection. Changes in sleep stage were found to be associated with changes in CMRO_2_ akin those observed in recent work from the authors’ lab in healthy subjects. The data showed, however, that the reduction in CMRO_2_ during deeper stages of sleep in OSA patients was greater than that found in healthy people, which is a consequence of arterial desaturation, a key hallmark of OSA. Desaturation over minutes of closely spaced apneas was prominent in a few subjects with severe forms of the disorder, where a string of closely spaced apneas caused a strong reduction in the arterio-venous difference from the combined increase in SvO_2_ and decrease in SaO_2_, not compensated by a commensurate increase in CBF (see Figures 6 and S1), resulting in reductions in CMRO_2_ by as much as 50–60%.

The particular implementation of UA-OxFlow involving a spiral readout in the OxFlow module (see Figure 2) has benefits beyond shortening overall cycle time, thereby yielding a temporal resolution of 5 seconds, not achievable with either Cartesian or radial sampling. However, most importantly, this design resulted in a reduction in acoustic noise by as much as 10 dB in the audible spectrum, which is critical given that the scanner environment is not conducive to sleep (see Fig. S2). However, spiral readout is more sensitive to field inhomogeneity than cartesian sampling, which can lead to discrepancies between the target and actual k-space trajectories. Agreement between trajectories was achieved with standard shimming at full width at half maximum (FWHM) values under 100 Hz. In the present study, FWHM of 50 Hz was typically achieved.

Although our initial results clearly indicate the feasibility of concurrently evaluating the acute cerebrovascular-metabolic implications of OSA paralleling detection of the site of airway collapse, some enhancements of the technology to facilitate successful execution of the protocol are envisioned. One of these is the uncertainty of prospectively positioning two axial imaging slices within the airway where airway closure is most common (retropalatal and retroglossal). Interleaved recording of a midline sagittal image through the airway mitigates this problem (see Figure 6) but will not enable quantification of cross-sectional airway area during apneas and hypopneas. While incorporation of a larger number of slice locations into the airway anatomic portion of the sequence is feasible, the trade-off would entail a reduction in temporal resolution. Alternatively, a more detailed examination of the likely sites of contact can be ascertained based on imaging the airway at high resolution prior to the UA-OxFlow scan prescription, which would provide information about the subject’s specific phenotype.^34,35^ Another potential obstacle to applying the method in large prospective studies is the limited capacity of the general population to endure scan protocols exceeding 30 minutes. Accustoming subjects to the MRI environment can be achieved by means of a mock scanner that simulates the MRI environment in terms of space constraints and acoustic noise. While further mitigation of scanner noise to the subject is achieved with MRI-compatible noise-canceling earphones, the current EEG set-up precludes their use. Even though further lessening acoustic noise may desirable, we found in this preliminary work that conducting the study during participants’ natural bedtime, between 9:30 and 11:30 pm, all subjects achieved and maintained sleep for a significant portion of the 90-minute protocol period permitting detection of apneas.

The present study was designed to determine the feasibility of detecting the neurometabolic changes of OSA patients during sleep. The results provide compelling evidence that the current method can detect the acute neurometabolic and airway changes during in-scanner sleep as a function of sleep stage in a protocol of duration comparable to an entire sleep cycle. The results further show that within our limited sample of OSA patients, temporal changes in neurometabolism occur in synchrony with hypopneas and apneas as indicated by changes in the cross-sectional area of the upper airway and in airflow as measured via nasal canula. Of note is that all 10 subjects enrolled in this preliminary sleep study achieved N1 sleep with nine of these having reached at least N2 sleep. Validation of these results requires much a larger sample size as there can be significant heterogeneity in the presentation of OSA.

## Conclusions

Concurrent evaluation of the dynamic changes in brain oxygen metabolism and airway architecture by quantitative MRI is feasible, both in healthy subjects and in patients with sleep apnea, yielding results in absolute physiological units at a rate of one full data set every 5-seconds. Concurrent EEG recording and upper airway airflow provide complementary information on sleep-stage dependence of apneas and hypopneas.

## Supplemental Material

sj-pdf-1-jcb-10.1177_0271678X251345293 - Supplemental material for Concurrent evaluation of cerebral oxygen metabolism and upper airway architecture via temporally resolved MRI Supplemental material, sj-pdf-1-jcb-10.1177_0271678X251345293 for Concurrent evaluation of cerebral oxygen metabolism and upper airway architecture via temporally resolved MRI by Jeffrey B Dennison, Michael C Langham, Andrew S Wiemken, Jing Xu, Richard J Schwab, John A Detre and Felix W Wehrli in Journal of Cerebral Blood Flow & Metabolism

## Data Availability

The data that support the findings of this study are included within the article and/or its supplementary materials.
