# Supplementary material for: Concurrent evaluation of cerebral oxygen metabolism and upper airway architecture via temporally resolved MRI
Source: J Cereb Blood Flow Metab. 2025 May 25;45(10):2047–59. doi: 10.1177/0271678X251345293 (PMC12106373; doi:10.1177/0271678X251345293)
Supplement: sj-pdf-1-jcb-10.1177_0271678X251345293 - Supplemental material for Concurrent evaluation of cerebral oxygen metabolism and upper airway architecture via temporally resolved MRI [file sj-pdf-1-jcb-10.1177_0271678X251345293.pdf]

Supplemental Material

Fig. S1 shows the time-course of sleep data in a 36-year-old male with severe apnea, displaying a pattern similar to that shown in Fig. 6, with steep decline in $CMRO_2$ during N2 sleep at around t=70 min.

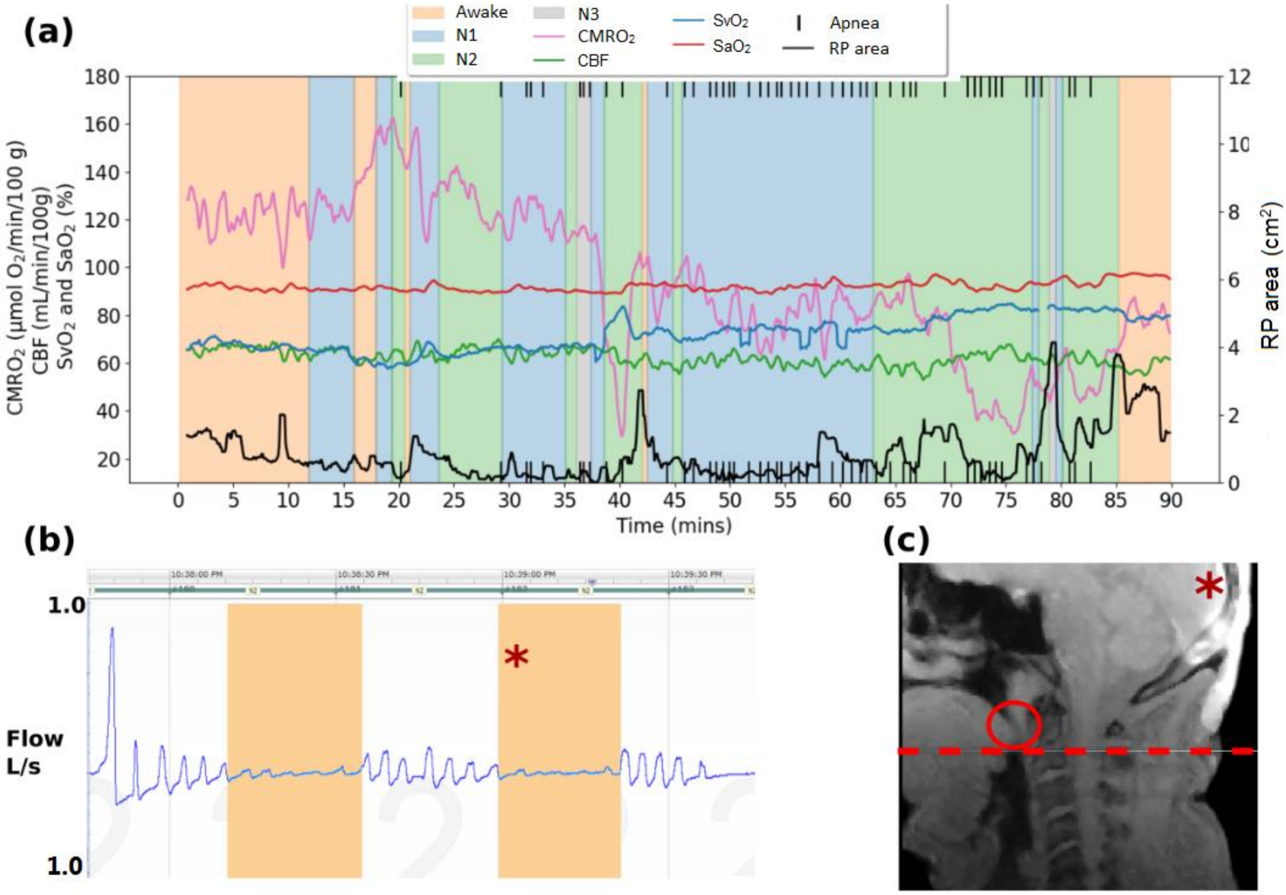

Figure S1. a) Data from a 36-year-old OSA patient (ID OSA-210) with an AHI of 29. The neurometabolic time-series is displayed with a carpet plot overlaid indicating the temporal location of apneas during the scan. $CMRO_2$ decreases from a baseline value of 130 during wakefulness to an average of about 80 (μmol O_2/min)/100g during N1, and finally to a nadir of approximately 40 (μmol O_2/min)/100g during N2 sleep. **b)** Plethysmographic recording of a series of closely spaced respiratory events showing a decrease in air flow during each successive event occurring over a period of two minutes. **c)** Sagittal airway image corresponding to the third respiratory event (asterisk) and contact between the retropalatal boundary and posterior airway margins. The axial slice location chosen prior to scanning is indicated by the red dashed line.

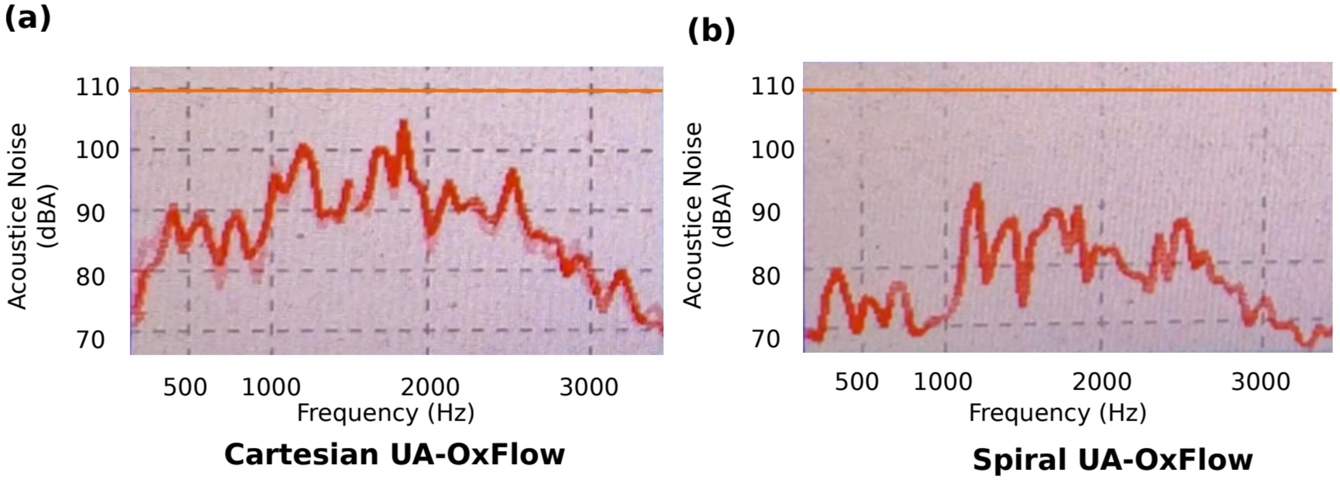

Figure S2. Acoustic power spectral density obtained with the OptoActive noise cancellation system (Optoacoustics, Israel), in decibels (dBA) for the frequency range most susceptible to the human ear: **a)** Cartesian, **b)** Spiral readout strategies of the UA-OxFlow MRI sequence. The orange line has been added to highlight the reduced acoustic noise across the entire spectrum for the spiral readout across the displayed spectrum.

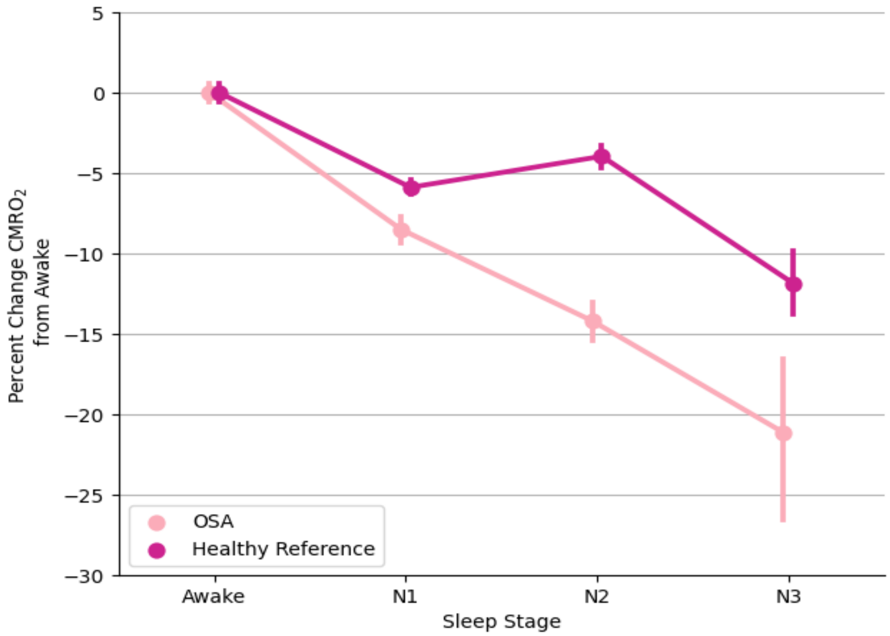

Figure S3. Average values of change in $CMRO_2$ from average awake values for each stage of sleep across four healthy reference subjects and six OSA patients. Differing levels of consciousness from awake to N3 (the deepest level of sleep observed in our sample) are plotted against average values of $CMRO_2$. A general trend across all subjects suggests that the sleep dependent changes in $CMRO_2$ are measurable via the spiral OxFlow sequence. Each point represents the mean change in $CMRO_2$ from each participant's average $CMRO_2$ during periods of wakefulness. Bars represent a 95% confidence interval for the mean value in percent change for each group from the waking condition.
